# Bis[μ-*N*-(3-meth­oxy-2-oxidobenzyl­idene-1:2κ^2^ *O* ^2^:*O* ^2^)-l-isoleucinato-2κ^2^ *N*,*O*]bis­(1,10-phenanthroline-1κ^2^ *N*,*N*′)dinickel(II) methanol tetra­solvate trihydrate

**DOI:** 10.1107/S1600536812008987

**Published:** 2012-03-03

**Authors:** Yan Li, Zhenghua Guo, Jianfang Dong, Lianzhi Li

**Affiliations:** a School of Chemistry and Chemical Engineering, Liaocheng University, Shandong 252059, People’s Republic of China

## Abstract

In the title complex, [Ni_2_(C_14_H_17_NO_4_)_2_(C_12_H_8_N_2_)_2_]·4CH_3_OH·3H_2_O, the two Ni^II^ ions are bridged by two Schiff base anions, leading to a dinuclear complex. One Ni^II^ ion is six-coordinated by four O atoms and two N atoms of two tridentate Schiff base ligands derived from the condensation of l-isoleucine and *o*-vanillin. The other Ni^II^ ion is six-coordinated by four N atoms of two 1,10-phenanthroline ligands and two O atoms of the Schiff base ligands. In the crystal, inter­molecular O—H⋯O and C—H⋯O hydrogen bonds lead to a three-dimensional structure. Intra­molecular C—H⋯O hydrogen bonds are also present. One of the methyl groups of the l-isoleucinate moieties is disordered over two sets of sites with an occupancy ratio of 0.687 (19):0.313 (19) and two methanol mol­ecules are half-occupied.

## Related literature

For transition metal compounds containing Schiff base ligands, see: Bernal *et al.* (1999 ▶); Chattopadhyay *et al.* (2009 ▶); Chohan *et al.* (1998 ▶).
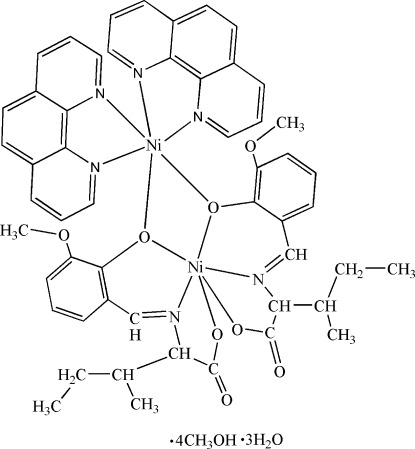


## Experimental

### 

#### Crystal data

[Ni_2_(C_14_H_17_NO_4_)_2_(C_12_H_8_N_2_)_2_]·4CH_4_O·3H_2_O
*M* *_r_* = 1186.62
Orthorhombic, 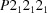

*a* = 14.1827 (15) Å
*b* = 14.3876 (16) Å
*c* = 28.787 (2) Å
*V* = 5874.1 (10) Å^3^
*Z* = 4
Mo *K*α radiation
μ = 0.71 mm^−1^
*T* = 298 K
0.50 × 0.36 × 0.35 mm

#### Data collection

Bruker SMART 1000 CCD diffractometer
Absorption correction: multi-scan (*SADABS*; Sheldrick, 1996 ▶) *T*_min_ = 0.718, *T*_max_ = 0.789
24389 measured reflections
10254 independent reflections
7528 reflections with *I* > 2σ(*I*)
*R*_int_ = 0.058

#### Refinement

*R*[*F* ^2^ > 2σ(*F* ^2^)] = 0.054
*wR*(*F* ^2^) = 0.157
*S* = 1.04
10254 reflections
740 parameters
H-atom parameters constrained
Δρ_max_ = 0.48 e Å^−3^
Δρ_min_ = −0.38 e Å^−3^
Absolute structure: Flack (1983 ▶), 4525 Friedel pairs
Flack parameter: 0.015 (16)

### 

Data collection: *SMART* (Bruker, 2007 ▶); cell refinement: *SAINT* (Bruker, 2007 ▶); data reduction: *SAINT*; program(s) used to solve structure: *SHELXS97* (Sheldrick, 2008 ▶); program(s) used to refine structure: *SHELXL97* (Sheldrick, 2008 ▶); molecular graphics: *SHELXTL* (Sheldrick, 2008 ▶); software used to prepare material for publication: *SHELXTL*.

## Supplementary Material

Crystal structure: contains datablock(s) global, I. DOI: 10.1107/S1600536812008987/hy2519sup1.cif

Structure factors: contains datablock(s) I. DOI: 10.1107/S1600536812008987/hy2519Isup2.hkl

Additional supplementary materials:  crystallographic information; 3D view; checkCIF report

## Figures and Tables

**Table 1:** Hydrogen-bond geometry (Å, °)

*D*—H⋯*A*	*D*—H	H⋯*A*	*D*⋯*A*	*D*—H⋯*A*
O9—H9⋯O1	0.82	1.88	2.693 (7)	169
O10—H10⋯O5	0.82	1.93	2.659 (7)	147
O11—H11⋯O10^i^	0.82	1.81	2.632 (10)	178
O12—H12⋯O15^ii^	0.82	1.97	2.75 (2)	158
O13—H13⋯O15	0.82	2.12	2.94 (3)	179
O14—H14*F*⋯O6	0.85	1.91	2.760 (8)	180
O14—H14*G*⋯O2^i^	0.85	2.05	2.901 (8)	180
O15—H15*C*⋯O9	0.85	1.85	2.690 (10)	169
O15—H15*D*⋯O16	0.85	2.06	2.901 (14)	171
O16—H16*C*⋯O11	0.85	1.97	2.817 (13)	178
O16—H16*D*⋯O14	0.85	1.95	2.798 (11)	178
C18—H18*B*⋯O6	0.97	2.52	3.192 (10)	126
C29—H29⋯O4	0.93	2.60	3.348 (8)	138
C30—H30⋯O12^iii^	0.93	2.57	3.405 (15)	149
C53—H53*C*⋯O6	0.96	2.53	3.460 (12)	164

Symmetry codes: (i) 
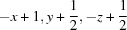
; (ii) 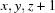; (iii) 
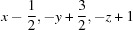
.

**Fig. 1.**
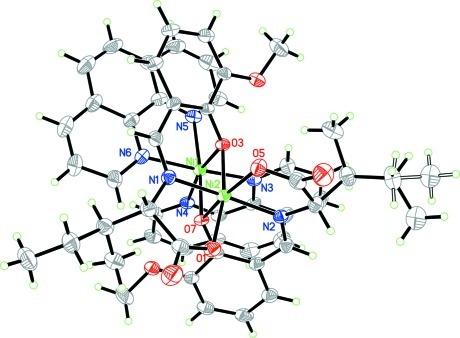
The molecular structure of the title compound, drawn with 30% probability displacement ellipsoids. Solvent molecules were omited for clarity.

## supplementary crystallographic information

### Comment

Transition metal compounds containing Schiff base ligands have been of great interest for many years (Bernal *et al.*, 1999; Chattopadhyay *et al.*, 2009). It has been reported that amino acid Schiff base and their first row transition metal complexes exhibit diverse biological activities (Chohan *et al.*, 1998). Herein, we report the synthesis and crystal structure of a binuclear nickel(II) complex with two tridentate Schiff base ligands derived from the condensation of L-isoleucine and *o*-vanillin and with two 1,10-phenanthroline coligands.

As shown in Fig. 1, both Ni^II^ ions are six-coordinated and reside in a distorted octahedral coordination environment. Ni2 atom is coordinated by two O atoms from carboxylate groups, two N atoms from imine groups and two O atoms from hydroxyl groups of the tridentate Schiff base ligands, forming a distorted octahedral coordination polyhedron. The axial bond angle, O7—Ni2—O5 [169.54 (15)°], is deviated from the ideal value 180°. N1, N2, O1 and O3 atoms are located in the equatorial plane, and Ni2 lies 0.032 (2) Å above the plane. Ni1 atom is also six-coordinated by four N atoms from two 1,10-phenanthroline ligands and two O atoms from hydroxyl groups of the tridentate Schiff base ligands, forming an octahedral geometry. The axial bond angle N6—Ni1—N3 is 169.6 (2)°, and Ni1 atom deviates 0.004 (2) Å from the equatorial plane formed by N4, N5, O3 and O7 atoms. In the crystal, intermolecular O—H···O and C—H···O hydrogen bonds lead to a three-dimensional structure (Table 1). Intramolecular C—H···O hydrogen bonds are present.

### Experimental

L-Isoleucine (1 mmol, 131.2 mg) and potassium hydroxide (1 mmol, 56.1 mg) were dissolved in hot methanol (10 ml) and added successively to a methanol solution of *o*-vanillin (1 mmol, 152.2 mg). The mixture was then stirred at 323 K for 2 h. Subsequently, an aqueous solution (2 ml) of nickel(II) chloride hexahydrate (1 mmol, 237.7 mg) was added dropwise and stirred for 2 h. A methanol solution (5 ml) of phenanthroline (1 mmol, 198.2 mg) was added dropwise and stirred for 4 h. The solution was held at room temperature for ten days, whereupon green blocky-shaped crystals suitable for X-ray diffraction were obtained.

### Refinement

Difference Fourier maps revealed that one of the methyl group of the Schiff base ligands is disordered over two sites. The subsequent refinement of their occupancies gave values of 0.687 (19) and 0.313 (19). H atoms of the water molecules were found in difference Fourier maps and refined as riding atoms, with O—H = 0.85 Å and with *U*_iso_(H) = 1.2*U*_eq_(O). Other H atoms were placed in geometrically calculated positions and refined as riding atoms, with C—H = 0.93 (aromatic), 0.96 (CH_3_), 0.97 (CH_2_) and 0.98 (CH) Å and O—H = 0.82 Å and with *U*_iso_(H) = 1.2(1.5 for methyl and hydroxyl)*U*_eq_(C,O).

### Crystal data

**Table:**

[Ni_2_(C_14_H_17_NO_4_)_2_(C_12_H_8_N_2_)_2_]·4CH_4_O·3H_2_O	*F*(000) = 2504
*M**_r_* = 1186.62	*D*_x_ = 1.342 Mg m^−^^3^
Orthorhombic, *P*2_1_2_1_2_1_	Mo *K*α radiation, λ = 0.71073 Å
Hall symbol: P 2ac 2ab	Cell parameters from 6251 reflections
*a* = 14.1827 (15) Å	θ = 2.1–22.3°
*b* = 14.3876 (16) Å	µ = 0.71 mm^−^^1^
*c* = 28.787 (2) Å	*T* = 298 K
*V* = 5874.1 (10) Å^3^	Block, green
*Z* = 4	0.50 × 0.36 × 0.35 mm

### Data collection

**Table:**

Bruker SMART 1000 CCD diffractometer	10254 independent reflections
Radiation source: fine-focus sealed tube	7528 reflections with *I* > 2σ(*I*)
Graphite monochromator	*R*_int_ = 0.058
φ and ω scans	θ_max_ = 25.0°, θ_min_ = 1.4°
Absorption correction: multi-scan (*SADABS*; Sheldrick, 1996)	*h* = −16→16
*T*_min_ = 0.718, *T*_max_ = 0.789	*k* = −17→14
24389 measured reflections	*l* = −34→27

### Refinement

**Table:**

Refinement on *F*^2^	Secondary atom site location: difference Fourier map
Least-squares matrix: full	Hydrogen site location: inferred from neighbouring sites
*R*[*F*^2^ > 2σ(*F*^2^)] = 0.054	H-atom parameters constrained
*wR*(*F*^2^) = 0.157	*w* = 1/[σ^2^(*F*_o_^2^) + (0.0843*P*)^2^ + 0.2853*P*] where *P* = (*F*_o_^2^ + 2*F*_c_^2^)/3
*S* = 1.04	(Δ/σ)_max_ = 0.001
10254 reflections	Δρ_max_ = 0.48 e Å^−^^3^
740 parameters	Δρ_min_ = −0.38 e Å^−^^3^
0 restraints	Absolute structure: Flack (1983), 4525 Friedel pairs
Primary atom site location: structure-invariant direct methods	Flack parameter: 0.015 (16)

### Special details

**Table:**

Geometry. All e.s.d.'s (except the e.s.d. in the dihedral angle between two l.s. planes) are estimated using the full covariance matrix. The cell e.s.d.'s are taken into account individually in the estimation of e.s.d.'s in distances, angles and torsion angles; correlations between e.s.d.'s in cell parameters are only used when they are defined by crystal symmetry. An approximate (isotropic) treatment of cell e.s.d.'s is used for estimating e.s.d.'s involving l.s. planes.
Refinement. Refinement of *F*^2^ against ALL reflections. The weighted *R*-factor *wR* and goodness of fit *S* are based on *F*^2^, conventional *R*-factors *R* are based on *F*, with *F* set to zero for negative *F*^2^. The threshold expression of *F*^2^ > σ(*F*^2^) is used only for calculating *R*-factors(gt) *etc*. and is not relevant to the choice of reflections for refinement. *R*-factors based on *F*^2^ are statistically about twice as large as those based on *F*, and *R*- factors based on ALL data will be even larger.

### Fractional atomic coordinates and isotropic or equivalent isotropic displacement parameters (Å^2^)

**Table:**

	*x*	*y*	*z*	*U*_iso_*/*U*_eq_	Occ. (<1)
Ni1	0.03559 (5)	0.36746 (5)	0.08944 (2)	0.03316 (19)	
Ni2	0.22288 (5)	0.42714 (5)	0.14628 (2)	0.03314 (19)	
N1	0.2127 (4)	0.3271 (3)	0.19494 (15)	0.0365 (11)	
N2	0.2567 (3)	0.5352 (3)	0.10536 (15)	0.0359 (11)	
N3	0.0359 (4)	0.4823 (3)	0.04619 (15)	0.0400 (11)	
N4	0.0179 (3)	0.3031 (3)	0.02394 (16)	0.0372 (11)	
N5	−0.1105 (3)	0.3774 (4)	0.10477 (16)	0.0416 (12)	
N6	0.0125 (4)	0.2470 (3)	0.12675 (16)	0.0420 (12)	
O1	0.3623 (3)	0.3827 (3)	0.14860 (14)	0.0430 (9)	
O2	0.4656 (4)	0.3041 (3)	0.19199 (18)	0.0682 (14)	
O3	0.0782 (3)	0.4453 (2)	0.14790 (12)	0.0325 (8)	
O4	−0.0674 (3)	0.5554 (3)	0.16137 (14)	0.0465 (10)	
O5	0.2534 (3)	0.5235 (3)	0.19671 (13)	0.0448 (11)	
O6	0.3347 (4)	0.6532 (3)	0.20795 (16)	0.0693 (14)	
O7	0.1841 (2)	0.3514 (2)	0.08909 (12)	0.0311 (8)	
O8	0.2101 (3)	0.2010 (3)	0.03901 (14)	0.0474 (11)	
O9	0.5038 (4)	0.4988 (4)	0.1256 (2)	0.0844 (17)	
H9	0.4600	0.4621	0.1289	0.127*	
O10	0.3015 (6)	0.4606 (4)	0.2803 (2)	0.105 (2)	
H10	0.2931	0.4587	0.2521	0.157*	
O11	0.7727 (7)	0.8424 (6)	0.1603 (3)	0.154 (3)	
H11	0.7494	0.8798	0.1784	0.232*	
O12	0.4841 (12)	0.6276 (10)	0.9727 (7)	0.141 (7)	0.50
H12	0.4871	0.6275	1.0011	0.212*	0.50
O13	0.742 (2)	0.6327 (17)	0.0328 (9)	0.223 (11)	0.50
H13	0.6873	0.6325	0.0416	0.335*	0.50
O14	0.4813 (5)	0.7793 (4)	0.2115 (2)	0.109 (2)	
H14F	0.4361	0.7405	0.2103	0.130*	
H14G	0.4969	0.7866	0.2398	0.130*	
O15	0.5444 (8)	0.6326 (6)	0.0635 (3)	0.192 (5)	
H15C	0.5303	0.5859	0.0802	0.231*	
H15D	0.5611	0.6766	0.0815	0.231*	
O16	0.5971 (7)	0.7692 (6)	0.1330 (3)	0.167 (4)	
H16C	0.6505	0.7911	0.1406	0.200*	
H16D	0.5611	0.7713	0.1565	0.200*	
C1	0.3831 (5)	0.3261 (4)	0.1805 (2)	0.0439 (15)	
C2	0.3014 (4)	0.2786 (4)	0.2056 (2)	0.0439 (16)	
H2	0.3126	0.2826	0.2392	0.053*	
C3	0.2970 (5)	0.1742 (4)	0.1918 (2)	0.0502 (16)	
H3	0.2325	0.1532	0.1980	0.060*	
C4	0.3624 (6)	0.1133 (4)	0.2210 (3)	0.067 (2)	
H4A	0.4268	0.1342	0.2166	0.080*	
H4B	0.3467	0.1215	0.2535	0.080*	
C5	0.3568 (7)	0.0099 (5)	0.2091 (3)	0.088 (3)	
H5A	0.2923	−0.0102	0.2104	0.133*	
H5B	0.3935	−0.0250	0.2311	0.133*	
H5C	0.3812	−0.0002	0.1784	0.133*	
C6	0.3138 (5)	0.1610 (5)	0.1398 (2)	0.064 (2)	
H6A	0.3797	0.1679	0.1331	0.095*	
H6B	0.2787	0.2069	0.1228	0.095*	
H6C	0.2934	0.1001	0.1307	0.095*	
C7	0.1443 (5)	0.3209 (4)	0.2234 (2)	0.0413 (15)	
H7	0.1523	0.2800	0.2480	0.050*	
C8	0.0538 (4)	0.3716 (4)	0.22165 (19)	0.0390 (13)	
C9	0.0301 (4)	0.4348 (3)	0.18687 (17)	0.0305 (11)	
C10	−0.0507 (4)	0.4890 (4)	0.19425 (18)	0.0364 (13)	
C11	−0.1120 (5)	0.4717 (4)	0.2304 (2)	0.0464 (15)	
H11A	−0.1675	0.5057	0.2330	0.056*	
C12	−0.0907 (5)	0.4033 (4)	0.2629 (2)	0.0530 (18)	
H12A	−0.1327	0.3898	0.2867	0.064*	
C13	−0.0077 (5)	0.3562 (4)	0.2594 (2)	0.0469 (16)	
H13A	0.0086	0.3132	0.2822	0.056*	
C14	−0.1357 (6)	0.6238 (5)	0.1692 (3)	0.074 (2)	
H14A	−0.1962	0.5949	0.1731	0.111*	
H14B	−0.1378	0.6653	0.1431	0.111*	
H14C	−0.1200	0.6580	0.1968	0.111*	
C15	0.2928 (5)	0.5965 (4)	0.1823 (2)	0.0474 (16)	
C16	0.2873 (5)	0.6193 (4)	0.13048 (19)	0.0444 (14)	
H16	0.3504	0.6367	0.1196	0.053*	
C17	0.2195 (6)	0.7028 (4)	0.1225 (2)	0.0573 (18)	
H17	0.1982	0.7003	0.0902	0.069*	
C18	0.2680 (7)	0.7962 (5)	0.1298 (3)	0.081 (3)	
H18A	0.2196	0.8439	0.1303	0.097*	0.687 (19)
H18B	0.2971	0.7955	0.1603	0.097*	0.687 (19)
H18C	0.2580	0.8134	0.1620	0.097*	0.313 (19)
H18D	0.3349	0.7839	0.1266	0.097*	0.313 (19)
C19	0.3402 (11)	0.8245 (9)	0.0959 (5)	0.104 (6)	0.687 (19)
H19A	0.3114	0.8330	0.0660	0.156*	0.687 (19)
H19B	0.3877	0.7771	0.0938	0.156*	0.687 (19)
H19C	0.3687	0.8817	0.1057	0.156*	0.687 (19)
C19'	0.248 (2)	0.8805 (19)	0.1030 (11)	0.104 (13)	0.313 (19)
H19D	0.2806	0.9322	0.1166	0.156*	0.313 (19)
H19E	0.1812	0.8924	0.1034	0.156*	0.313 (19)
H19F	0.2684	0.8722	0.0715	0.156*	0.313 (19)
C20	0.1311 (5)	0.6957 (5)	0.1540 (3)	0.069 (2)	
H20A	0.1490	0.7057	0.1858	0.104*	
H20B	0.1035	0.6351	0.1508	0.104*	
H20C	0.0860	0.7420	0.1449	0.104*	
C21	0.2769 (5)	0.5277 (4)	0.06221 (19)	0.0400 (14)	
H21	0.3022	0.5802	0.0480	0.048*	
C22	0.2648 (4)	0.4470 (4)	0.03308 (19)	0.0396 (14)	
C23	0.2283 (4)	0.3625 (4)	0.04890 (17)	0.0325 (12)	
C24	0.2382 (4)	0.2849 (4)	0.0198 (2)	0.0387 (14)	
C25	0.2713 (5)	0.2932 (4)	−0.0254 (2)	0.0504 (16)	
H25	0.2748	0.2412	−0.0445	0.061*	
C26	0.2984 (5)	0.3781 (5)	−0.0415 (2)	0.0559 (18)	
H26	0.3183	0.3839	−0.0722	0.067*	
C27	0.2972 (5)	0.4540 (4)	−0.0139 (2)	0.0494 (16)	
H27	0.3175	0.5110	−0.0255	0.059*	
C28	0.2374 (6)	0.1167 (4)	0.0173 (3)	0.076 (2)	
H28A	0.2051	0.1105	−0.0118	0.114*	
H28B	0.2214	0.0653	0.0371	0.114*	
H28C	0.3043	0.1173	0.0120	0.114*	
C29	0.0400 (5)	0.5701 (4)	0.0581 (2)	0.0498 (15)	
H29	0.0394	0.5849	0.0895	0.060*	
C30	0.0453 (5)	0.6436 (5)	0.0254 (3)	0.0638 (19)	
H30	0.0469	0.7052	0.0351	0.077*	
C31	0.0478 (6)	0.6215 (6)	−0.0201 (3)	0.071 (2)	
H31	0.0529	0.6686	−0.0420	0.085*	
C32	0.0427 (5)	0.5284 (5)	−0.0350 (2)	0.0522 (17)	
C33	0.0360 (4)	0.4603 (4)	0.00005 (19)	0.0407 (14)	
C34	0.0267 (4)	0.3665 (4)	−0.01187 (18)	0.0397 (13)	
C35	0.0277 (5)	0.3383 (5)	−0.0591 (2)	0.0480 (16)	
C36	0.0147 (5)	0.2456 (5)	−0.0686 (2)	0.0577 (19)	
H36	0.0126	0.2251	−0.0992	0.069*	
C37	0.0050 (5)	0.1833 (5)	−0.0328 (2)	0.0546 (18)	
H37	−0.0031	0.1203	−0.0390	0.066*	
C38	0.0071 (4)	0.2149 (5)	0.0128 (2)	0.0501 (17)	
H38	0.0007	0.1717	0.0366	0.060*	
C39	0.0459 (6)	0.4992 (6)	−0.0822 (2)	0.064 (2)	
H39	0.0535	0.5431	−0.1056	0.077*	
C40	0.0381 (5)	0.4105 (6)	−0.0930 (2)	0.065 (2)	
H40	0.0394	0.3940	−0.1242	0.078*	
C41	−0.1719 (5)	0.4423 (5)	0.0922 (2)	0.0553 (17)	
H41	−0.1530	0.4899	0.0724	0.066*	
C42	−0.2679 (5)	0.4399 (6)	0.1089 (3)	0.068 (2)	
H42	−0.3112	0.4843	0.0992	0.082*	
C43	−0.2943 (5)	0.3719 (6)	0.1392 (3)	0.067 (2)	
H43	−0.3560	0.3711	0.1502	0.080*	
C44	−0.2335 (5)	0.3057 (5)	0.1537 (2)	0.0571 (18)	
C45	−0.1407 (4)	0.3098 (4)	0.1348 (2)	0.0425 (15)	
C46	−0.0745 (5)	0.2405 (4)	0.1462 (2)	0.0456 (15)	
C47	−0.1021 (6)	0.1689 (5)	0.1767 (2)	0.0577 (19)	
C48	−0.0346 (7)	0.1003 (5)	0.1856 (3)	0.070 (2)	
H48	−0.0478	0.0528	0.2065	0.084*	
C49	0.0508 (6)	0.1030 (5)	0.1637 (3)	0.067 (2)	
H49	0.0941	0.0553	0.1681	0.081*	
C50	0.0728 (5)	0.1782 (4)	0.1345 (2)	0.0552 (18)	
H50	0.1315	0.1798	0.1201	0.066*	
C51	−0.2548 (6)	0.2340 (6)	0.1859 (3)	0.072 (2)	
H51	−0.3140	0.2327	0.1998	0.086*	
C52	−0.1928 (6)	0.1693 (6)	0.1967 (3)	0.072 (2)	
H52	−0.2092	0.1231	0.2178	0.086*	
C53	0.5307 (8)	0.5333 (7)	0.1689 (4)	0.116 (4)	
H53A	0.5763	0.5820	0.1648	0.175*	
H53B	0.5581	0.4841	0.1870	0.175*	
H53C	0.4765	0.5575	0.1848	0.175*	
C54	0.3889 (8)	0.4915 (8)	0.2891 (3)	0.109 (4)	
H54A	0.3912	0.5576	0.2844	0.163*	
H54B	0.4328	0.4618	0.2685	0.163*	
H54C	0.4053	0.4775	0.3207	0.163*	
C55	0.8155 (14)	0.8919 (10)	0.1217 (6)	0.223 (9)	
H55A	0.8651	0.9312	0.1331	0.334*	
H55B	0.7686	0.9293	0.1066	0.334*	
H55C	0.8410	0.8480	0.1000	0.334*	
C56	0.5384 (17)	0.5598 (15)	0.9558 (8)	0.116 (7)	0.50
H56A	0.6028	0.5803	0.9550	0.174*	0.50
H56B	0.5181	0.5442	0.9250	0.174*	0.50
H56C	0.5334	0.5060	0.9754	0.174*	0.50
C57	0.781 (2)	0.7202 (19)	0.0402 (11)	0.168 (12)	0.50
H57A	0.7398	0.7561	0.0595	0.252*	0.50
H57B	0.7892	0.7511	0.0109	0.252*	0.50
H57C	0.8413	0.7138	0.0552	0.252*	0.50

### Atomic displacement parameters (Å^2^)

**Table:**

	*U*^11^	*U*^22^	*U*^33^	*U*^12^	*U*^13^	*U*^23^
Ni1	0.0339 (4)	0.0375 (4)	0.0281 (3)	0.0004 (3)	0.0014 (3)	0.0002 (3)
Ni2	0.0324 (4)	0.0388 (4)	0.0283 (4)	0.0000 (3)	0.0011 (3)	0.0012 (3)
N1	0.038 (3)	0.038 (3)	0.034 (3)	0.000 (2)	0.000 (3)	0.0031 (19)
N2	0.037 (3)	0.041 (2)	0.030 (2)	−0.001 (2)	0.003 (2)	−0.0023 (19)
N3	0.039 (3)	0.047 (3)	0.034 (3)	0.001 (2)	0.001 (2)	−0.002 (2)
N4	0.029 (3)	0.046 (3)	0.036 (3)	0.000 (2)	−0.001 (2)	−0.005 (2)
N5	0.031 (3)	0.054 (3)	0.039 (3)	0.000 (3)	−0.003 (2)	−0.012 (2)
N6	0.042 (3)	0.046 (3)	0.038 (3)	−0.007 (2)	0.000 (2)	0.004 (2)
O1	0.034 (2)	0.053 (2)	0.042 (2)	0.0045 (19)	0.001 (2)	0.010 (2)
O2	0.039 (3)	0.083 (3)	0.083 (4)	0.005 (3)	−0.006 (3)	0.028 (3)
O3	0.034 (2)	0.039 (2)	0.0240 (18)	0.0015 (16)	0.0018 (18)	−0.0017 (16)
O4	0.044 (3)	0.048 (2)	0.047 (2)	0.0108 (19)	0.002 (2)	−0.0060 (19)
O5	0.054 (3)	0.046 (2)	0.034 (2)	−0.011 (2)	0.000 (2)	−0.0010 (17)
O6	0.080 (4)	0.069 (3)	0.058 (3)	−0.020 (3)	−0.007 (3)	−0.019 (2)
O7	0.026 (2)	0.042 (2)	0.0248 (18)	0.0016 (16)	0.0024 (17)	−0.0029 (17)
O8	0.054 (3)	0.044 (2)	0.044 (2)	0.002 (2)	0.010 (2)	−0.0108 (19)
O9	0.060 (4)	0.090 (4)	0.103 (5)	−0.010 (3)	0.003 (3)	0.002 (3)
O10	0.141 (7)	0.105 (5)	0.069 (4)	−0.003 (5)	−0.018 (4)	0.007 (3)
O11	0.174 (9)	0.157 (7)	0.133 (7)	0.041 (7)	0.002 (6)	−0.007 (6)
O12	0.152 (15)	0.081 (9)	0.191 (17)	−0.046 (10)	−0.047 (13)	0.043 (10)
O13	0.22 (3)	0.21 (2)	0.24 (3)	0.03 (2)	−0.02 (2)	0.10 (2)
O14	0.111 (6)	0.112 (5)	0.102 (5)	−0.039 (4)	−0.020 (4)	0.004 (4)
O15	0.207 (11)	0.162 (8)	0.207 (10)	−0.013 (8)	0.037 (9)	0.072 (8)
O16	0.199 (10)	0.158 (8)	0.144 (8)	−0.043 (7)	0.024 (7)	0.007 (6)
C1	0.040 (4)	0.048 (3)	0.044 (4)	0.005 (3)	−0.003 (3)	−0.002 (3)
C2	0.042 (4)	0.047 (4)	0.043 (4)	0.010 (3)	−0.001 (3)	0.008 (3)
C3	0.047 (4)	0.050 (4)	0.055 (4)	0.005 (3)	−0.001 (3)	−0.001 (3)
C4	0.066 (5)	0.056 (4)	0.078 (5)	0.015 (4)	−0.003 (4)	0.015 (4)
C5	0.087 (7)	0.056 (5)	0.123 (8)	0.022 (4)	0.000 (6)	0.008 (5)
C6	0.063 (5)	0.066 (5)	0.062 (5)	0.009 (4)	0.002 (4)	−0.010 (4)
C7	0.050 (4)	0.039 (3)	0.035 (3)	0.005 (3)	0.001 (3)	0.006 (3)
C8	0.044 (4)	0.039 (3)	0.034 (3)	−0.004 (3)	0.008 (3)	−0.002 (3)
C9	0.031 (3)	0.035 (3)	0.025 (3)	0.000 (3)	0.002 (2)	−0.003 (2)
C10	0.038 (4)	0.038 (3)	0.033 (3)	0.001 (3)	0.002 (3)	−0.008 (2)
C11	0.044 (4)	0.052 (4)	0.043 (3)	0.001 (3)	0.007 (3)	−0.006 (3)
C12	0.049 (4)	0.064 (4)	0.046 (4)	0.003 (3)	0.020 (3)	−0.001 (3)
C13	0.053 (4)	0.051 (4)	0.037 (3)	0.000 (3)	0.013 (3)	0.004 (3)
C14	0.081 (6)	0.061 (5)	0.080 (5)	0.026 (4)	0.002 (4)	−0.009 (4)
C15	0.050 (4)	0.047 (4)	0.045 (4)	0.002 (3)	−0.005 (3)	−0.011 (3)
C16	0.047 (4)	0.045 (3)	0.041 (3)	−0.002 (3)	0.000 (3)	0.003 (3)
C17	0.067 (5)	0.048 (4)	0.056 (4)	−0.004 (4)	−0.009 (4)	−0.008 (3)
C18	0.099 (7)	0.049 (4)	0.095 (6)	−0.003 (4)	−0.009 (6)	−0.003 (4)
C19	0.105 (13)	0.076 (9)	0.130 (13)	−0.012 (8)	0.023 (10)	−0.002 (9)
C19'	0.11 (3)	0.08 (2)	0.13 (3)	−0.012 (18)	0.02 (2)	−0.002 (18)
C20	0.066 (5)	0.074 (5)	0.068 (5)	0.015 (4)	0.000 (4)	−0.019 (4)
C21	0.042 (4)	0.041 (3)	0.037 (3)	0.001 (3)	0.001 (3)	0.007 (2)
C22	0.040 (4)	0.048 (3)	0.031 (3)	0.002 (3)	0.005 (3)	0.002 (2)
C23	0.027 (3)	0.043 (3)	0.028 (3)	0.006 (3)	−0.002 (2)	0.001 (2)
C24	0.033 (3)	0.045 (3)	0.038 (3)	0.002 (3)	0.000 (3)	−0.003 (3)
C25	0.053 (4)	0.058 (4)	0.040 (3)	0.009 (3)	0.003 (3)	−0.013 (3)
C26	0.063 (5)	0.072 (5)	0.032 (3)	0.014 (4)	0.013 (3)	−0.005 (3)
C27	0.051 (4)	0.056 (4)	0.041 (3)	0.002 (3)	0.006 (3)	0.000 (3)
C28	0.099 (7)	0.051 (4)	0.078 (5)	0.014 (4)	0.011 (5)	−0.023 (4)
C29	0.054 (4)	0.049 (4)	0.047 (4)	0.001 (4)	−0.006 (3)	−0.001 (3)
C30	0.070 (5)	0.053 (4)	0.068 (5)	0.001 (4)	−0.017 (4)	0.009 (4)
C31	0.074 (6)	0.079 (5)	0.059 (5)	−0.011 (5)	−0.010 (4)	0.025 (4)
C32	0.050 (4)	0.066 (4)	0.040 (3)	−0.005 (4)	−0.006 (3)	0.012 (3)
C33	0.029 (3)	0.058 (4)	0.035 (3)	−0.003 (3)	0.000 (3)	0.009 (3)
C34	0.031 (3)	0.057 (4)	0.030 (3)	0.002 (3)	0.000 (3)	−0.002 (3)
C35	0.037 (4)	0.074 (5)	0.033 (3)	−0.002 (3)	−0.004 (3)	−0.003 (3)
C36	0.049 (4)	0.083 (5)	0.042 (4)	0.012 (4)	−0.003 (3)	−0.013 (4)
C37	0.052 (4)	0.059 (4)	0.052 (4)	0.000 (3)	−0.003 (3)	−0.023 (3)
C38	0.043 (4)	0.053 (4)	0.055 (4)	−0.002 (3)	−0.002 (3)	−0.009 (3)
C39	0.067 (5)	0.082 (5)	0.044 (4)	−0.005 (4)	−0.005 (4)	0.016 (4)
C40	0.062 (5)	0.101 (6)	0.031 (3)	−0.003 (4)	−0.005 (4)	−0.005 (4)
C41	0.049 (4)	0.068 (4)	0.049 (4)	0.005 (4)	−0.007 (3)	−0.017 (3)
C42	0.045 (4)	0.084 (5)	0.076 (5)	0.007 (4)	−0.011 (4)	−0.029 (4)
C43	0.043 (4)	0.085 (5)	0.073 (5)	−0.012 (4)	0.011 (4)	−0.018 (4)
C44	0.043 (4)	0.068 (4)	0.060 (4)	−0.008 (4)	0.005 (4)	−0.017 (3)
C45	0.037 (4)	0.054 (4)	0.036 (3)	−0.011 (3)	0.000 (3)	−0.011 (3)
C46	0.048 (4)	0.053 (4)	0.036 (3)	−0.014 (3)	0.004 (3)	−0.007 (3)
C47	0.065 (5)	0.061 (4)	0.047 (4)	−0.022 (4)	0.002 (4)	0.002 (3)
C48	0.081 (6)	0.065 (5)	0.062 (5)	−0.024 (5)	−0.001 (5)	0.017 (3)
C49	0.076 (6)	0.056 (4)	0.071 (5)	−0.010 (4)	−0.012 (4)	0.020 (3)
C50	0.057 (4)	0.052 (4)	0.056 (4)	0.000 (3)	−0.004 (3)	0.008 (3)
C51	0.054 (5)	0.088 (6)	0.073 (5)	−0.028 (5)	0.025 (4)	−0.008 (4)
C52	0.075 (6)	0.078 (5)	0.061 (5)	−0.028 (5)	0.014 (5)	0.006 (4)
C53	0.094 (8)	0.120 (8)	0.136 (10)	0.010 (7)	0.022 (8)	−0.031 (7)
C54	0.117 (10)	0.112 (8)	0.097 (8)	−0.003 (7)	−0.037 (7)	0.005 (6)
C55	0.25 (2)	0.207 (18)	0.207 (18)	−0.056 (16)	0.059 (16)	0.063 (14)
C56	0.108 (18)	0.102 (16)	0.139 (19)	0.002 (15)	0.013 (16)	−0.027 (14)
C57	0.18 (3)	0.15 (2)	0.17 (3)	0.00 (2)	0.03 (2)	0.05 (2)

### Geometric parameters (Å, º)

**Table:**

Ni1—N6	2.065 (5)	C18—C19'	1.47 (3)
Ni1—N3	2.069 (5)	C18—C19	1.472 (16)
Ni1—O3	2.110 (3)	C18—H18A	0.9700
Ni1—N4	2.115 (5)	C18—H18B	0.9700
Ni1—O7	2.119 (4)	C18—H18C	0.9699
Ni1—N5	2.123 (5)	C18—H18D	0.9701
Ni2—N2	2.009 (4)	C19—H19A	0.9600
Ni2—N1	2.014 (4)	C19—H19B	0.9600
Ni2—O7	2.049 (3)	C19—H19C	0.9600
Ni2—O5	2.053 (4)	C19'—H19D	0.9600
Ni2—O3	2.069 (4)	C19'—H19E	0.9600
Ni2—O1	2.080 (4)	C19'—H19F	0.9600
N1—C7	1.271 (7)	C20—H20A	0.9600
N1—C2	1.471 (7)	C20—H20B	0.9600
N2—C21	1.279 (7)	C20—H20C	0.9600
N2—C16	1.475 (7)	C21—C22	1.443 (8)
N3—C29	1.310 (7)	C21—H21	0.9300
N3—C33	1.365 (7)	C22—C23	1.397 (8)
N4—C38	1.318 (7)	C22—C27	1.432 (8)
N4—C34	1.382 (7)	C23—C24	1.403 (7)
N5—C41	1.328 (8)	C24—C25	1.388 (8)
N5—C45	1.370 (8)	C25—C26	1.362 (9)
N6—C50	1.327 (8)	C25—H25	0.9300
N6—C46	1.359 (8)	C26—C27	1.350 (8)
O1—C1	1.263 (7)	C26—H26	0.9300
O2—C1	1.257 (7)	C27—H27	0.9300
O3—C9	1.322 (6)	C28—H28A	0.9600
O4—C10	1.365 (7)	C28—H28B	0.9600
O4—C14	1.400 (8)	C28—H28C	0.9600
O5—C15	1.260 (7)	C29—C30	1.416 (9)
O6—C15	1.250 (7)	C29—H29	0.9300
O7—C23	1.326 (6)	C30—C31	1.349 (10)
O8—C24	1.386 (7)	C30—H30	0.9300
O8—C28	1.418 (7)	C31—C32	1.408 (10)
O9—C53	1.396 (11)	C31—H31	0.9300
O9—H9	0.8200	C32—C33	1.409 (8)
O10—C54	1.341 (12)	C32—C39	1.424 (9)
O10—H10	0.8200	C33—C34	1.399 (8)
O11—C55	1.452 (14)	C34—C35	1.419 (8)
O11—H11	0.8200	C35—C36	1.373 (9)
O12—C56	1.33 (2)	C35—C40	1.434 (9)
O12—H12	0.8200	C36—C37	1.373 (10)
O13—C57	1.39 (3)	C36—H36	0.9300
O13—H13	0.8200	C37—C38	1.389 (9)
O14—H14F	0.8499	C37—H37	0.9300
O14—H14G	0.8502	C38—H38	0.9300
O15—H15C	0.8499	C39—C40	1.318 (10)
O15—H15D	0.8500	C39—H39	0.9300
O16—H16C	0.8501	C40—H40	0.9300
O16—H16D	0.8499	C41—C42	1.444 (10)
C1—C2	1.527 (9)	C41—H41	0.9300
C2—C3	1.555 (8)	C42—C43	1.361 (11)
C2—H2	0.9800	C42—H42	0.9300
C3—C6	1.528 (9)	C43—C44	1.351 (10)
C3—C4	1.529 (9)	C43—H43	0.9300
C3—H3	0.9800	C44—C51	1.421 (10)
C4—C5	1.528 (10)	C44—C45	1.424 (9)
C4—H4A	0.9700	C45—C46	1.409 (9)
C4—H4B	0.9700	C46—C47	1.408 (9)
C5—H5A	0.9600	C47—C48	1.399 (11)
C5—H5B	0.9600	C47—C52	1.410 (11)
C5—H5C	0.9600	C48—C49	1.365 (11)
C6—H6A	0.9600	C48—H48	0.9300
C6—H6B	0.9600	C49—C50	1.406 (9)
C6—H6C	0.9600	C49—H49	0.9300
C7—C8	1.477 (8)	C50—H50	0.9300
C7—H7	0.9300	C51—C52	1.317 (11)
C8—C9	1.394 (7)	C51—H51	0.9300
C8—C13	1.411 (8)	C52—H52	0.9300
C9—C10	1.402 (8)	C53—H53A	0.9600
C10—C11	1.379 (8)	C53—H53B	0.9600
C11—C12	1.391 (9)	C53—H53C	0.9600
C11—H11A	0.9300	C54—H54A	0.9600
C12—C13	1.362 (9)	C54—H54B	0.9600
C12—H12A	0.9300	C54—H54C	0.9600
C13—H13A	0.9300	C55—H55A	0.9600
C14—H14A	0.9600	C55—H55B	0.9600
C14—H14B	0.9600	C55—H55C	0.9600
C14—H14C	0.9600	C56—H56A	0.9600
C15—C16	1.531 (8)	C56—H56B	0.9600
C16—C17	1.555 (9)	C56—H56C	0.9600
C16—H16	0.9800	C57—H57A	0.9600
C17—C18	1.524 (9)	C57—H57B	0.9600
C17—C20	1.551 (10)	C57—H57C	0.9600
C17—H17	0.9800		
			
N6—Ni1—N3	169.6 (2)	C19—C18—H18D	46.1
N6—Ni1—O3	94.35 (16)	C17—C18—H18D	105.5
N3—Ni1—O3	93.18 (16)	H18A—C18—H18D	145.3
N6—Ni1—N4	94.45 (18)	H18B—C18—H18D	70.5
N3—Ni1—N4	79.25 (18)	H18C—C18—H18D	106.2
O3—Ni1—N4	167.74 (16)	C18—C19—H19A	109.5
N6—Ni1—O7	93.95 (18)	H18D—C19—H19A	141.9
N3—Ni1—O7	94.69 (17)	C18—C19—H19B	109.5
O3—Ni1—O7	77.10 (13)	H18D—C19—H19B	73.2
N4—Ni1—O7	93.78 (16)	H19A—C19—H19B	109.5
N6—Ni1—N5	78.1 (2)	C18—C19—H19C	109.5
N3—Ni1—N5	94.2 (2)	H18D—C19—H19C	104.7
O3—Ni1—N5	94.47 (16)	H19A—C19—H19C	109.5
N4—Ni1—N5	95.70 (18)	H19B—C19—H19C	109.5
O7—Ni1—N5	168.04 (16)	C18—C19'—H19D	109.5
N2—Ni2—N1	168.06 (19)	C18—C19'—H19E	109.5
N2—Ni2—O7	90.26 (16)	H19D—C19'—H19E	109.5
N1—Ni2—O7	99.18 (16)	C18—C19'—H19F	109.5
N2—Ni2—O5	80.89 (16)	H19D—C19'—H19F	109.5
N1—Ni2—O5	90.34 (17)	H19E—C19'—H19F	109.5
O7—Ni2—O5	169.54 (15)	C17—C20—H20A	109.5
N2—Ni2—O3	98.77 (17)	C17—C20—H20B	109.5
N1—Ni2—O3	90.18 (17)	H20A—C20—H20B	109.5
O7—Ni2—O3	79.56 (14)	C17—C20—H20C	109.5
O5—Ni2—O3	96.19 (16)	H20A—C20—H20C	109.5
N2—Ni2—O1	91.70 (17)	H20B—C20—H20C	109.5
N1—Ni2—O1	80.01 (18)	N2—C21—C22	127.3 (5)
O7—Ni2—O1	96.76 (15)	N2—C21—H21	116.4
O5—Ni2—O1	89.11 (17)	C22—C21—H21	116.4
O3—Ni2—O1	168.89 (15)	C23—C22—C27	119.2 (5)
C7—N1—C2	119.0 (5)	C23—C22—C21	123.7 (5)
C7—N1—Ni2	123.5 (4)	C27—C22—C21	117.0 (5)
C2—N1—Ni2	115.0 (4)	O7—C23—C22	124.3 (5)
C21—N2—C16	118.6 (5)	O7—C23—C24	118.2 (5)
C21—N2—Ni2	123.9 (4)	C22—C23—C24	117.5 (5)
C16—N2—Ni2	114.7 (3)	O8—C24—C25	123.1 (5)
C29—N3—C33	118.5 (5)	O8—C24—C23	115.2 (5)
C29—N3—Ni1	127.8 (4)	C25—C24—C23	121.6 (5)
C33—N3—Ni1	113.6 (4)	C26—C25—C24	119.5 (6)
C38—N4—C34	117.7 (5)	C26—C25—H25	120.3
C38—N4—Ni1	130.7 (4)	C24—C25—H25	120.3
C34—N4—Ni1	111.4 (3)	C27—C26—C25	121.4 (6)
C41—N5—C45	117.8 (6)	C27—C26—H26	119.3
C41—N5—Ni1	129.1 (5)	C25—C26—H26	119.3
C45—N5—Ni1	112.9 (4)	C26—C27—C22	120.2 (6)
C50—N6—C46	117.7 (5)	C26—C27—H27	119.9
C50—N6—Ni1	127.7 (4)	C22—C27—H27	119.9
C46—N6—Ni1	114.7 (4)	O8—C28—H28A	109.5
C1—O1—Ni2	116.3 (4)	O8—C28—H28B	109.5
C9—O3—Ni2	121.1 (3)	H28A—C28—H28B	109.5
C9—O3—Ni1	117.9 (3)	O8—C28—H28C	109.5
Ni2—O3—Ni1	101.48 (15)	H28A—C28—H28C	109.5
C10—O4—C14	120.0 (5)	H28B—C28—H28C	109.5
C15—O5—Ni2	115.1 (4)	N3—C29—C30	123.3 (6)
C23—O7—Ni2	120.7 (3)	N3—C29—H29	118.3
C23—O7—Ni1	117.5 (3)	C30—C29—H29	118.3
Ni2—O7—Ni1	101.85 (15)	C31—C30—C29	118.0 (7)
C24—O8—C28	119.3 (5)	C31—C30—H30	121.0
C53—O9—H9	109.5	C29—C30—H30	121.0
C54—O10—H10	109.5	C30—C31—C32	121.2 (7)
C55—O11—H11	109.5	C30—C31—H31	119.4
C56—O12—H12	109.5	C32—C31—H31	119.4
C57—O13—H13	109.5	C31—C32—C33	116.6 (6)
H14F—O14—H14G	108.4	C31—C32—C39	124.8 (6)
H15C—O15—H15D	108.2	C33—C32—C39	118.7 (6)
H16C—O16—H16D	108.3	N3—C33—C34	117.5 (5)
O2—C1—O1	124.8 (6)	N3—C33—C32	122.4 (5)
O2—C1—C2	118.0 (5)	C34—C33—C32	120.1 (5)
O1—C1—C2	117.2 (5)	N4—C34—C33	117.5 (5)
N1—C2—C1	109.7 (5)	N4—C34—C35	121.8 (6)
N1—C2—C3	111.7 (5)	C33—C34—C35	120.7 (5)
C1—C2—C3	109.9 (5)	C36—C35—C34	117.9 (6)
N1—C2—H2	108.5	C36—C35—C40	125.6 (6)
C1—C2—H2	108.5	C34—C35—C40	116.5 (6)
C3—C2—H2	108.5	C37—C36—C35	119.9 (6)
C6—C3—C4	111.9 (6)	C37—C36—H36	120.0
C6—C3—C2	111.4 (5)	C35—C36—H36	120.0
C4—C3—C2	112.9 (5)	C36—C37—C38	119.5 (6)
C6—C3—H3	106.7	C36—C37—H37	120.3
C4—C3—H3	106.7	C38—C37—H37	120.3
C2—C3—H3	106.7	N4—C38—C37	123.3 (7)
C5—C4—C3	113.8 (7)	N4—C38—H38	118.4
C5—C4—H4A	108.8	C37—C38—H38	118.4
C3—C4—H4A	108.8	C40—C39—C32	120.6 (6)
C5—C4—H4B	108.8	C40—C39—H39	119.7
C3—C4—H4B	108.8	C32—C39—H39	119.7
H4A—C4—H4B	107.7	C39—C40—C35	123.3 (6)
C4—C5—H5A	109.5	C39—C40—H40	118.3
C4—C5—H5B	109.5	C35—C40—H40	118.3
H5A—C5—H5B	109.5	N5—C41—C42	120.7 (7)
C4—C5—H5C	109.5	N5—C41—H41	119.6
H5A—C5—H5C	109.5	C42—C41—H41	119.6
H5B—C5—H5C	109.5	C43—C42—C41	119.3 (8)
C3—C6—H6A	109.5	C43—C42—H42	120.3
C3—C6—H6B	109.5	C41—C42—H42	120.3
H6A—C6—H6B	109.5	C44—C43—C42	121.9 (7)
C3—C6—H6C	109.5	C44—C43—H43	119.1
H6A—C6—H6C	109.5	C42—C43—H43	119.1
H6B—C6—H6C	109.5	C43—C44—C51	125.3 (7)
N1—C7—C8	127.4 (5)	C43—C44—C45	116.3 (7)
N1—C7—H7	116.3	C51—C44—C45	118.4 (7)
C8—C7—H7	116.3	N5—C45—C46	116.2 (6)
C9—C8—C13	120.4 (5)	N5—C45—C44	124.0 (6)
C9—C8—C7	123.8 (5)	C46—C45—C44	119.8 (6)
C13—C8—C7	115.8 (5)	N6—C46—C47	124.1 (6)
O3—C9—C8	124.0 (5)	N6—C46—C45	117.3 (5)
O3—C9—C10	119.1 (5)	C47—C46—C45	118.6 (6)
C8—C9—C10	116.9 (5)	C48—C47—C46	116.1 (7)
O4—C10—C11	122.7 (5)	C48—C47—C52	123.6 (7)
O4—C10—C9	115.2 (5)	C46—C47—C52	120.3 (7)
C11—C10—C9	121.9 (5)	C49—C48—C47	120.2 (7)
C10—C11—C12	119.8 (6)	C49—C48—H48	119.9
C10—C11—H11A	120.1	C47—C48—H48	119.9
C12—C11—H11A	120.1	C48—C49—C50	119.6 (7)
C13—C12—C11	119.4 (6)	C48—C49—H49	120.2
C13—C12—H12A	120.3	C50—C49—H49	120.2
C11—C12—H12A	120.3	N6—C50—C49	122.1 (7)
C12—C13—C8	120.9 (6)	N6—C50—H50	118.9
C12—C13—H13A	119.6	C49—C50—H50	118.9
C8—C13—H13A	119.6	C52—C51—C44	121.7 (7)
O4—C14—H14A	109.5	C52—C51—H51	119.1
O4—C14—H14B	109.5	C44—C51—H51	119.1
H14A—C14—H14B	109.5	C51—C52—C47	121.0 (7)
O4—C14—H14C	109.5	C51—C52—H52	119.5
H14A—C14—H14C	109.5	C47—C52—H52	119.5
H14B—C14—H14C	109.5	O9—C53—H53A	109.5
O6—C15—O5	124.2 (6)	O9—C53—H53B	109.5
O6—C15—C16	117.3 (6)	H53A—C53—H53B	109.5
O5—C15—C16	118.4 (5)	O9—C53—H53C	109.5
N2—C16—C15	108.5 (5)	H53A—C53—H53C	109.5
N2—C16—C17	112.3 (5)	H53B—C53—H53C	109.5
C15—C16—C17	109.9 (5)	O10—C54—H54A	109.5
N2—C16—H16	108.7	O10—C54—H54B	109.5
C15—C16—H16	108.7	H54A—C54—H54B	109.5
C17—C16—H16	108.7	O10—C54—H54C	109.5
C18—C17—C20	110.0 (6)	H54A—C54—H54C	109.5
C18—C17—C16	112.4 (6)	H54B—C54—H54C	109.5
C20—C17—C16	111.3 (5)	O11—C55—H55A	109.5
C18—C17—H17	107.6	O11—C55—H55B	109.5
C20—C17—H17	107.6	H55A—C55—H55B	109.5
C16—C17—H17	107.6	O11—C55—H55C	109.5
C19'—C18—C19	63.8 (14)	H55A—C55—H55C	109.5
C19'—C18—C17	124.7 (14)	H55B—C55—H55C	109.5
C19—C18—C17	117.8 (8)	O12—C56—H56A	109.5
C19—C18—H18A	107.9	O12—C56—H56B	109.5
C17—C18—H18A	107.9	H56A—C56—H56B	109.5
C19'—C18—H18B	124.6	O12—C56—H56C	109.5
C19—C18—H18B	107.9	H56A—C56—H56C	109.5
C17—C18—H18B	107.9	H56B—C56—H56C	109.5
H18A—C18—H18B	107.2	O13—C57—H57A	109.5
C19'—C18—H18C	105.2	O13—C57—H57B	109.5
C19—C18—H18C	131.7	H57A—C57—H57B	109.5
C17—C18—H18C	106.9	O13—C57—H57C	109.5
H18A—C18—H18C	72.7	H57A—C57—H57C	109.5
C19'—C18—H18D	107.0	H57B—C57—H57C	109.5
			
C1—C2—C3—C4	85.0 (7)		

### Hydrogen-bond geometry (Å, º)

**Table:**

*D*—H···*A*	*D*—H	H···*A*	*D*···*A*	*D*—H···*A*
O9—H9···O1	0.82	1.88	2.693 (7)	169
O10—H10···O5	0.82	1.93	2.659 (7)	147
O11—H11···O10^i^	0.82	1.81	2.632 (10)	178
O12—H12···O15^ii^	0.82	1.97	2.75 (2)	158
O13—H13···O15	0.82	2.12	2.94 (3)	179
O14—H14*F*···O6	0.85	1.91	2.760 (8)	180
O14—H14*G*···O2^i^	0.85	2.05	2.901 (8)	180
O15—H15*C*···O9	0.85	1.85	2.690 (10)	169
O15—H15*D*···O16	0.85	2.06	2.901 (14)	171
O16—H16*C*···O11	0.85	1.97	2.817 (13)	178
O16—H16*D*···O14	0.85	1.95	2.798 (11)	178
C18—H18*B*···O6	0.97	2.52	3.192 (10)	126
C29—H29···O4	0.93	2.60	3.348 (8)	138
C30—H30···O12^iii^	0.93	2.57	3.405 (15)	149
C53—H53*C*···O6	0.96	2.53	3.460 (12)	164

Symmetry codes: (i) −*x*+1, *y*+1/2, −*z*+1/2; (ii) *x*, *y*, *z*+1; (iii) *x*−1/2, −*y*+3/2, −*z*+1.
